# Combination of Early EEG, Brain CT, and Ammonia Level Is Useful to Predict Neurologic Outcome in Children Resuscitated From Cardiac Arrest

**DOI:** 10.3389/fped.2019.00223

**Published:** 2019-06-04

**Authors:** Donghwa Yang, Eell Ryoo, Hyo Jeong Kim

**Affiliations:** Department of Pediatrics, Gachon University Gil Medical Center, Gachon University College of Medicine, Incheon, South Korea

**Keywords:** cardiac arrest, pediatric, neurologic outcome, electroencephalography, gray matter to white matter attenuation ratio, ammonia

## Abstract

**Purpose:** Predicting neurologic prognosis in pediatric patients recovered after cardiac arrest is more difficult than in adults. This study hypothesized that a combination model of early electroencephalography, brain computed tomography (CT), and laboratory findings improve prediction performance of neurologic outcome in pediatric patients after cardiac arrest.

**Methods:** We retrospectively analyzed the medical records of pediatric patients resuscitated after non-traumatic cardiac arrest. Clinical features, electroencephalography, gray matter to white matter attenuation ratio on brain CT, and laboratory findings were analyzed. The primary outcome was neurologic prognosis based on the Pediatric Cerebral Performance Category score.

**Results:** Of 21 patients, seven (33.3%) were classified as a good neurologic outcome group and 14 (66.7%) were classified as a poor neurologic outcome group. The good outcome group was associated with a slow and disorganized electroencephalographic background pattern (*P* = 0.006), reactivity (*P* = 0.006), and electrographic seizures (*P* = 0.03). The frequency of a suppressed electroencephalographic background pattern was significantly higher in the poor outcome group (*P* = 0.006). The poor outcome group was also associated with a low level of gray matter to white matter attenuation ratio (*P* = 0.03) and hyperammonemia (*P* = 0.003). The area under curve of the combined model, consisting of electroencephalographic background, gray matter to white matter attenuation ratio, and ammonia was the highest at 0.959 (0.772–0.999) with a specificity of 100%.

**Conclusion:** Unfavorable electroencephalographic background, low gray matter to white matter attenuation ratio on brain CT, and hyperammonemia are associated with poor neurologic outcome in children after cardiac arrest.

## Introduction

The annual incidence rate of out of hospital cardiac arrest worldwide varies from 2.6 to 19.7 children per 100,000 (1, 2) and the Korean annual incidence rate is 4.2 children per 100,000 (3). Approximately 47–69% of the patients resuscitated from cardiac arrest remain comatose and do not regain consciousness (4, 5). So, predicting the prognosis in children who suffer cardiac arrest is difficult. In adults, an unfavorable electroencephalography (EEG) pattern such as burst-suppression at 24 h after return of spontaneous circulation (ROSC), no light reflex at 48 h, and the absence of somatosensory evoked potential at 72 h are predictive of a poor prognosis (6). In studies on pediatric patients, neurologic outcome is good if the early EEG background is normal or if only slowing EEG background is present (7). Structural changes on brain computed tomography (CT) are known to be associated with a poor neurologic prognosis (8). Hyperammonemia is also known as one of signs of poor neurologic outcome (9).

However, the criteria for prediction of prognosis in pediatric patients with cardiac arrest are not clear. The purpose of this study is to investigate the usefulness of early EEG, brain CT and laboratory findings in predicting neurological outcomes in pediatric ROSC patients after non-traumatic cardiac arrest. In addition, we investigated the neurological prognostic performance of combined models of single variables.

## Materials and Methods

### Subjects and Study Design

We retrospectively analyzed the medical records of pediatric patients resuscitated after non-traumatic cardiac arrest at the Gachon University Gil Medical Center from January 2006 to December 2017. Patients between 1 month and 18 years of age were included. Cardiopulmonary resuscitation (CPR) was performed according to the Pediatric Advanced Life Support guidelines at that time during the course of the study (10–12). Patients who were dead on arrival, patients who did not have a CT scan within 24 h, patients who did not undergo EEG within 72 h, and patients who went to other hospitals during treatment were excluded from the study.

### Data Collection and Analysis

**Figure 1:**
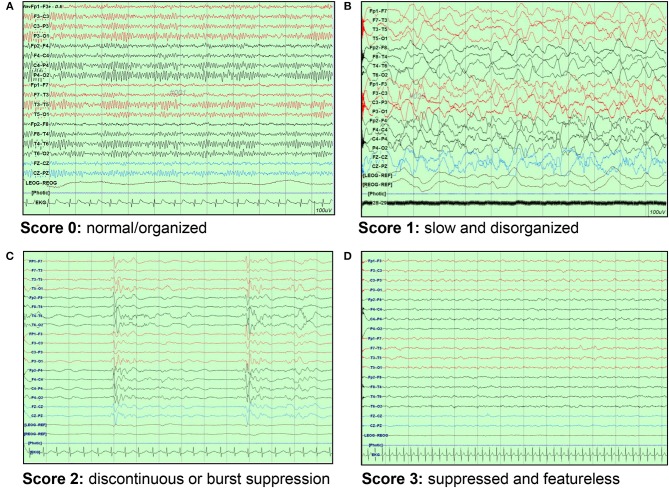
EEG background scoring. **(A)** Score 0: normal/organized pattern **(B)** Score 1: slow and disorganized pattern **(C)** Score 2: discontinuous or burst suppression pattern **(D)** Score 3: suppressed and featureless pattern. EEG, electroencephalography.

We reviewed each patient's medical records retrospectively and collected data on age, sex, arrest etiology, presence of underlying disease, EEG, drugs used during EEG, brain CT imaging, and laboratory findings. EEGs were performed according to international standard 10-20 system for 30 min as early as possible after ROSC. One pediatric neurologist analyzed the EEGs. EEGs were divided into having a score of 0 (normal/organized), 1 (slow and disorganized), 2 (discontinuous or burst suppression), and 3 (suppressed and featureless), and the criterion for suppression was < 10 μV (Figure 1) (13). The presence of reactivity and electrographic seizures were also investigated.

In addition, we evaluated the gray matter to white matter attenuation ratio (GWR) on brain CT images after measuring the Hounsfield unit (HU). Brain CT was performed using Sensation 16 or Definition Edge CT unit (Siemens, Erlangen, Germany), and one pediatric neurologist analyzed the images using image-viewing software (INFINITT PACS, Seoul, South Korea). With reference to previous studies reported by Metter (14), the HU values of the caudate nucleus (CN), putamen (PU), genu of the corpus callosum (CC), and posterior limb of the internal capsule (PIC) were obtained at the basal ganglia level of the brain CT. The GWR value was calculated by dividing the HU value measured in gray matter by the HU value in white matter (GWR = [CN + PU]/[CC + PIC]). On the brain CT images, HU values were obtained by dividing the total sum of the region of interest of 0.1–0.15 cm^2^ by area. Laboratory findings included ammonia, S-100, liver function tests, and blood gas analysis which were done in the emergency room. We selected the first results if there were multiple measurements including EEG, brain CT, and laboratory findings.

The primary outcome was assessed as a neurologic outcome at least 6 months after cardiac arrest. The neurologic outcome was evaluated according to the Pediatric Cerebral Performance Category (PCPC) as a score of 1 (normal), 2 (mild disability), 3 (moderate disability), 4 (severe disability), 5 (coma), and 6 (brain death) (15). PCPC score was determined based on the information in the medical record. Patients were divided into two groups: the good neurologic outcome group (PCPC 1–3) and the poor neurologic outcome group (PCPC 4–6).

This study was approved by the Institutional Review Board of Gachon University Gil Medical Center.

### Statistical Analysis

Statistical analyses were computed using MedCalc Ver. 18.2.1 (MedCalc Software bvba, Ostend, Belgium). To compare the mean of two groups, the Student's *t*-test or Mann–Whitney *U*-test was used for normal distribution. The Chi-square test or Fisher's exact test was used for categorical variables. To evaluate the neurological prognostic performance of each test, sensitivity, specificity, and area under the curve (AUC) were calculated by receiver operating characteristic (ROC). Logistic regression analysis was used for the AUC of the combined model with two or more variables. *P* < 0.05 were considered to be statistically significant.

## Results

### Patient Characteristics

Total 203 out-of-hospital cardiac arrest patients aged 1 month to 18 years visited the hospital. Of 203 patients, 132 patients who were deceased on arrival, 42 patients who did not undergo an EEG within 72 h, and eight patients who did not have a CT performed within 24 h were excluded. Ultimately, 21 patients (10.3%) were included in the study.

According to the PCPC score, there were seven patients (33.3%) with good neurological prognosis and 14 patients (66.7%) with poor neurological prognosis. The median age for the good outcome group was 0.5 years (interquartile ranges [IQR] 0.3–12.0) and the median age of the poor outcome group was 1.2 years (IQR 0.3–9.1). There was no significant difference in age between the two groups (*P* = 0.88). The mean time of cardiac arrest was 18.1 ± 9.0 min in the poor outcome group and 11.4 ± 9.3 min in the good outcome group, but there was no statistically significant difference (*P* = 0.13). The most common cause of cardiac arrest was asphyxia in both groups (good outcome group, 71.4% and poor outcome group, 64.3%); there was no difference in underlying disease in both groups (*P* = 0.54). Therapeutic hypothermia was performed in 9 patients. There was no difference in time from ROSC to EEG evaluation between the two groups (*P* = 0.85). The rates of using muscle relaxant (*P* = 0.62), anticonvulsant (*P* = 0.35), and sedative drugs (*P* = 0.34) were similar between the groups during the EEGs (Table 1).

**Table 1:** Clinical characteristics and neurologic outcomes.

**Characteristic**	**Good neurologic outcome** **(*n* = 7)**	**Poor neurologic outcome** **(*n* = 14)**	***P*-value**
Age in years, median (IQR)	0.5 (0.3–12.0)	1.2 (0.3–9.1)	0.88
Male sex, *n* (%)	6 (85.7)	8 (57.1)	0.34
Arrest duration in minutes, mean ± SD	11.4 ± 9.3	18.1 ± 9.0	0.13
Arrest etiology, *n* (%)			1.00
Asphyxia/Respiratory	5 (71.4)	9 (64.3)	1.00
Cardiac	0	1 (7.1)	1.00
Near drowning	0	1 (7.1)	1.00
Others	2 (28.6)	3 (21.4)	1.00
Preexisting conditions, *n* (%)			0.54
None	4 (57.1)	10 (71.4)	0.64
Neurologic	0	2 (14.3)	0.53
Cardiac	1 (14.3)	1 (7.1)	1.00
Other	2 (28.6)	1 (7.1)	0.25
Initial cardiac rhythm, *n* (%)			1.00
PEA	1 (14.3)	2 (14.3)	1.00
V fib/Tachycardia	0	1 (7.1)	1.00
Asystole	6 (85.7)	11 (78.6)	1.00
Hypothermia treatment, *n* (%)	3 (42.9)	6 (42.9)	1.00
ROSC to EEG onset, *n* (%)			0.85
< 24 h	3 (42.9)	7 (50.0)	1.00
Between 24 and ≤ 48 h	3 (42.9)	4 (28.6)	0.64
Between 48 and ≤ 72 h	1 (14.3)	3 (21.4)	1.00
Medication during EEG recording, *n* (%)			
Sedative drug			0.34
Fentanyl	1 (14.3)	1 (7.1)	1.00
Remifentanil	1 (14.3)	3 (21.4)	1.00
Midazolam	4 (57.1)	4 (28.6)	0.35
Muscle relaxant			0.62
Vecuronium	0	2 (14.3)	0.53
Rocuronium	1 (14.3)	2 (14.3)	1.00
Antiepileptic drug			0.35
Phenobarbital	3 (42.9)	2 (14.3)	0.28
Phenytoin	2 (28.6)	2 (14.3)	0.57
Valproic acid	1 (14.3)	1 (7.1)	1.00

IQR, interquartile ranges; SD, standard deviation; ROSC, return of spontaneous circulation; EEG, electroencephalography; PEA, pulseless electrical activity; V fib, ventricular fibrillation.

### Relationship Between EEG Findings and Neurological Outcome

The ratio of the slow and disorganized pattern (71.4%) in the EEG background was significantly higher in the good outcome group (*P* = 0.006), and the ratio of the suppressed and featureless pattern (92.9%) was higher in the poor outcome group (*P* = 0.006). Patients in the good outcome group were more likely to have more reactivity to stimulation than those in the poor outcome group (*P* = 0.006). Notably, electrographic seizures were significantly more common in the good outcome group except if there was status epilepticus (*P* = 0.03) (Table 2).

**Table 2:** EEG findings and neurologic outcomes.

**Finding**	**Good neurologic** **outcome (*n* = 7)**	**Poor neurologic outcome (*n* = 14)**	***P*-value**
EEG background score, *n* (%)			0.006
Score 0 (normal/organized)	0	0	NA
Score 1 (slow and disorganized)	5 (71.4)	1 (7.1)	0.006
Score 2 (discontinuous or burst suppression)	0	0	NA
Score 3 (suppressed and featureless)	2 (28.6)	13 (92.9)	0.006
Reactivity, *n* (%)	5 (71.4)	1 (7.1)	0.006
Electrographic seizures, *n* (%)			0.04
None	4 (57.1)	12 (85.7)	0.28
Seizure	3 (42.9)	0	0.03
Status epilepticus	0	2 (14.3)	0.53

EEG, electroencephalography; NA, not available.

### Prognostic Values of GWR on Brain CT and Laboratory Findings

The mean value of GWR measured at the basal ganglia level on brain CT was 1.20 ± 0.09 in the good outcome group and significantly higher than 1.11 ± 0.07 in the poor outcome group (*P* = 0.03).

In the laboratory findings, the pH was significantly lower in the poor outcome group than in the good outcome group (*P* = 0.006) and the lactate level was significantly higher in the poor outcome group (*P* = 0.006). The serum ammonia level was markedly elevated in the poor outcome group (mean value: 422 μg/dL [IQR 236–905]) than good outcome group (mean value: 83 μg/dL [IQR 68–268]) (*P* = 0.003) (Table 3).

**Table 3:** Brain CT, laboratory findings, and neurologic outcomes.

**Characteristic**	**Good neurologic outcome (*n* = 7)**	**Poor neurologic outcome (*n* = 14)**	***P*-value**
GWR in brain CT, mean ± SD	1.20 ± 0.09	1.11 ± 0.07	0.03
Laboratory findings, median (IQR)			
S-100 (μg/L) (*n* = 16)	1.0 (0.7–1.1)	1.3 (0.8–1.9)	0.19
AST (U/L) (*n* = 21)	70 (42–245)	238 (128–337)	0.08
ALT (U/L) (*n* = 21)	36 (19–218)	143 (58–201)	0.13
pH (*n* = 21)	7.13 (6.95–7.38)	6.8 (6.8–6.85)	0.006
Lactate (mmol/L) (*n* = 21)	4.4 (3.5–13.6)	15.0 (11.8–15.0)	0.006
Ammonia (μg/dL) (*n* = 21)	83 (68–268)	422 (236–905)	0.003

CT, computed tomography; GWR, gray matter to white matter attenuation ratio; SD, standard deviation; IQR, interquartile ranges; AST, aspartate transaminase; ALT, alanine transaminase.

### Combined Model of Single Predictors Using ROC Curve

ROC analyses were performed to investigate the predictive performance of the single variable model and multimodal models of neurological prognosis (Table 4). The EEG evaluation showed that favorable EEG background categories (score 0, 1) were associated with good outcome and the AUC was 0.821 (95% confidence interval [CI] 0.595–0.952) (*P* = 0.001). Unfavorable EEG background categories (score 2, 3) were associated with the poor outcome group (*P* = 0.001) and the AUC was 0.821 (0.595–0.952), with a specificity of 92.9% and a positive predictive value of 83.4%. Low GWR values on brain CTs predicted poor prognosis with an AUC of 0.776 (0.543–0.926) (*P* = 0.01), a cutoff value of 1.13 with a 71.4% specificity, and a cutoff value of 1.08 with 100% specificity. Hyperammonemia was predictive of poor prognosis (*P* < 0.001), with 71.4% specificity at a cutoff value of 147 μg/dL and 100% specificity at 334 μg/dL; the AUC was 0.888 (0.674–0.982).

**Table 4:** Predictive values for neurologic outcome.

**Variable**	**Predicted outcome**	**AUC**	**Sensitivity**	**Specificity**	**PPV**	**NPV**	***P*-value**
EEG background score 0, 1	Good	0.821 (0.595–0.952)	71.4 (29.0–96.3)	92.9 (66.1–99.8)	83.4 (41.6–97.3)	86.7 (66.7–95.5)	0.001
EEG background score 2, 3	Poor	0.821 (0.595–0.952)	71.4 (29.0–96.3)	92.9 (66.1–99.8)	83.4 (41.6–97.3)	86.7 (66.7–95.5)	0.001
Reactivity	Good	0.821 (0.595–0.952)	71.4 (29.0–96.3)	92.9 (66.1–99.8)	83.4 (41.6–97.3)	86.7 (66.7–95.5)	0.001
Electrographic seizures	Good	0.714 (0.478–0.887)	42.9 (9.9–81.6)	100 (76.8–100)	100	77.8 (64.9–87.0)	0.03
Brain CT GWR	Poor	0.776 (0.543–0.926)	71.4 (41.9–91.6)	71.4 (29.0–96.3)	83.3 (59.7–94.4)	55.5 (32.5–76.3)	0.01
Ammonia	Poor	0.888 (0.674–0.982)	92.9 (66.1–9.8)	71.4 (29.0–96.3)	86.6 (66.6–95.5)	83.5 (41.7–97.3)	<0.001
EEG background 2, 3 + Ammonia	Poor	0.929 (0.728–0.995)	92.9 (66.1–99.8)	85.7 (42.1–99.6)	92.8 (67.8–98.8)	85.8 (47.1–97.6)	<0.001
EEG background 2, 3 + Brain CT GWR	Poor	0.939 (0.742–0.997)	92.9 (66.1–99.8)	100 (59.0–100)	100	87.6 (51.5–97.9)	<0.001
EEG background 2, 3 + Brain CT GWR + Ammonia	Poor	0.959 (0.772–0.999)	92.9 (66.1–99.8)	100 (59.0–100)	100	87.6 (51.5–97.9)	<0.001

*Data are expressed as value (95% confidence interval). AUC, area under the curve; PPV, positive predictive value; NPV, negative predictive value; EEG, electroencephalography; GWR, gray matter to white matter attenuation ratio*.

**Figure 2:**
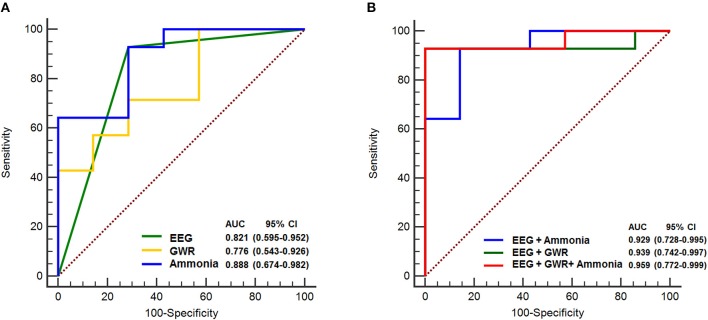
ROC curves to predict poor neurologic outcome. **(A)** AUC for single variable, EEG background score 2, 3: 0.821, GWR: 0.776, Ammonia: 0.888 **(B)** AUC for combination of EEG and Ammonia: 0.929, EEG and GWR: 0.939, EEG and GWR and Ammonia: 0.959. ROC, receiver operating characteristic; AUC, area under curve; EEG, electroencephalography; GWR, gray matter to white matter attenuation ratio; CI, confidence interval.

For predicting poor neurologic outcome, the combination of unfavorable EEG background (score 2, 3) and GWR had an AUC of 0.939 (0.742–0.997) and was higher than the AUC of 0.929 (0.728–0.995) for the combination of unfavorable EEG background associated with ammonia. Finally, the combination of the three parameters of unfavorable EEG background, GWR, and ammonia had the greatest AUC (0.959 [0.772–0.999]), with 100% specificity and positive predictive value (Table 4; Figure 2).

## Discussion

Brain damage in patients following cardiac arrest is affected not only by hypoxic-ischemic brain injuries that occur during cardiac arrest but also by reperfusion after resuscitation and recovery of blood circulation (16, 17). With this mechanism, multiple organs suffer from ischemic and reperfusion injury, which is called post-cardiac arrest syndrome (18). The survival rate of out-of-hospital cardiac arrest in pediatric patients is low, ranging from 3.3 to 15.8% (10). Even after survival, post-cardiac arrest syndrome can result in various sequelae. To determine the future treatment of patients who have recovered after cardiac arrest, many studies attempting to predict neurological prognosis have been performed. Neurological examinations are useful methods, but prognostication based on neurological examinations should be delayed in cases of hypothermic treatment (19). In contrast, EEG is known to be largely unaffected by hypothermia (20), and laboratory findings such as ammonia, and brain CT are advantageous in that they can be performed quickly and easily when the patients arrive at the emergency room.

In previous studies on EEG and neurologic prognosis in pediatric patients after cardiac arrest, an unfavorable EEG or absence of the reactivity predicted poor prognosis (21), and favorable background predicted a good prognosis (7).

This study showed that patients had neurologically good outcomes when there was a slow and disorganized background pattern, and in cases of presence of a response to stimulation, whereas neurologically poor prognosis when there was a background of a suppressed and featureless pattern. The suppressed and featureless pattern of the EEG background reflects the severity of the brain damage and the loss of brain function. However, it should also be considered that sedative drugs administered during hypothermic treatment and ventilator application can affect EEG background. In this study, the frequency of using sedative drugs, muscle relaxants, and anticonvulsants was not different between the two prognosis groups. Topjian et al. reported that electrographic seizures were associated with good neurological prognosis (21). In this study, electrographic seizures were also associated with good neurological prognosis except for status epilepticus. Two patients with status epilepticus had a poor prognosis in spite of using active anticonvulsants and sedatives. Rundgren et al. also reported poor neurological prognosis in the presence of status epilepticus on EEG (22).

The advantage of brain CT is that it can be performed early when the patient arrives at the hospital and can help to identify the etiology. Torbey et al. proposed a method for quantitatively representing boundary loss by measuring gray and white HU values on brain CTs (23). Metter et al. reported that the reduction in GWR values obtained from the equation (CN + PU)/(CC + PIC) at the basal ganglia level was associated with poor neurological prognosis (14). The mechanism by which the GWR value decreases in cardiac arrest is that the HU value of the gray matter decreases due to cytotoxic brain edema and neuronal necrosis when ischemic injury is present and the HU value increases due to the expansion effect of the vein entering into the white matter (24). Although studies have shown that patients with a GWR of < 1.18 to <1.22 can predict the specificity of 100% of deaths in adults (14, 23, 25), little has been done regarding the GWR values in children. Starling et al. reported that a GWR of < 1.14 is predictive of death in pediatric patients, but the specificity was not 100% (8). In our study, the GWR cutoff value of 1.08 at the basal ganglia level showed 100% specificity for poor prognosis prediction and showed the highest specificity and sensitivity when the cutoff value was 1.13. The lower cutoff value of GWR in children is due to the change in density of gray matter and white matter, probably associated with the normal myelination process (26, 27). In addition, attenuated CT protocol in pediatric patients might impact on the HU. However, we could not analyze the effect in detail.

Laboratory findings associated with the prognosis of cardiac arrest have been extensively studied in adult and pediatric patients, including S-100 and pH, but little has been studied regarding ammonia. Yanagawa et al. reported that hyperammonemia can predict poor prognosis with low blood pH in adult patients (9). This is because metabolic acidosis or respiratory acidosis leads to the release of ammonia from red blood cells. In this study, the median serum ammonia was 83 μg/dL (68–268) in the good outcome group, and 422 μg/dL (236–905) in the poor outcome group. Cutoff value of 334 μg/dL showed specificity of 100%.

Since the proposed prognostic predictor can improve the accuracy of the prognostic performance in the multimodal model, the prediction of the prognosis is suggested by combining various variables (6, 28–30). However, these multimodal models are limited to adult studies, and no studies have been reported on multimodal models of children. In this study, the AUC was calculated as 0.959 (0.772–0.999), which had a 100% specificity and positive predictive value, by combining early EEG findings, GWR on brain CT, and serum ammonia.

The limitation of this study is that it was a retrospective study of a small number of patients in a single institution. Patients who had an EEG within 72 h were included even in patients who underwent hypothermic treatment, which can delay the timing of an EEG. Due to the small number of patients, it was impossible to analyze the prognosis according to the timing of the EEG.

In conclusion, the suppressed and featureless patterns of EEG in pediatric patients resuscitated from out-of-hospital cardiac arrest were significantly higher in the poor prognosis group, and reactivity to stimulation and electrographic seizures, except for status epilepticus, were higher in the good prognosis group. Additionally, low GWR values on brain CT, lactic acidosis, and hyperammonemia were also significantly higher in the poor prognosis group. Unfavorable EEG background, low GWR on brain CT, and hyperammonemia are associated with poor neurological outcome in children resuscitated after an out-of-hospital cardiac arrest. In addition, the combined model of these prognosis predictors showed high specificity.

## Data Availability

All datasets generated for this study are included in the manuscript and/or the supplementary files.

## Ethics Statement

Institutional Review Board of Gachon University Gil Medical Center.

## Author Contributions

HK contributed conception and design of the study. DY and HK collected, analyzed, and interpreted the data, wrote and reviewed the manuscript. DY and ER performed the statistical analysis and reviewed the manuscript. All authors contributed to manuscript revision, read and approved the submitted version.

### Conflict of Interest Statement

The authors declare that the research was conducted in the absence of any commercial or financial relationships that could be construed as a potential conflict of interest.
